# Determinants and long‐term costs of early reproduction in males of a long‐lived polygynous mammal

**DOI:** 10.1002/ece3.7530

**Published:** 2021-04-08

**Authors:** Yanny Ritchot, Marco Festa‐Bianchet, David Coltman, Fanie Pelletier

**Affiliations:** ^1^ Département de biologie Université de Sherbrooke Sherbrooke QC Canada; ^2^ Department of Biological Sciences University of Alberta Edmonton AB Canada

**Keywords:** age at first reproduction, intraspecific competition, life history, male reproductive success, *Ovis canadensis*, polygyny

## Abstract

In long‐lived polygynous species, male reproductive success is often monopolized by a few mature dominant individuals. Young males are generally too small to be dominant and may employ alternative tactics; however, little is known about the determinants of reproductive success for young males. Understanding the causes and consequences of variability in early reproductive success may be crucial to assess the strength of sexual selection and possible long‐term trade‐offs among life‐history traits. Selective pressures driven by fluctuating environmental conditions may depend on age class. We evaluated the determinants of reproduction in male bighorn sheep (*Ovis canadensis*) aged 2–4 years using 30 years of individual‐level data. These young males cannot defend estrous ewes and use alternative mating tactics. We also investigated how the age of first detected reproduction was correlated to lifetime reproductive success and longevity. We found that reproductive success of males aged 3 years was positively correlated to body mass, to the proportion of males aged 2–4 years in the competitor pool, and to the number of females available per adult male. These results suggest that reproductive success depends on both competitive ability and population age–sex structure. None of these variables, however, had significant effects on the reproductive success of males aged 2 or 4 years. Known reproduction before the age of five increased lifetime reproductive success but decreased longevity, suggesting a long‐term survival cost of early reproduction. Our analyses reveal that both individual‐level phenotypic and population‐level demographic variables influence reproductive success by young males and provide a rare assessment of fitness trade‐offs in wild polygynous males.

## Graphical abstract

Little is known about the determinants of reproductive success for young polygynous male mammals, although the causes and consequences of variability in early reproductive success may be crucial to assess the strength of sexual selection and possible long‐term trade‐offs among life‐history traits. We evaluated the determinants of reproduction in male bighorn sheep (*Ovis canadensis*) aged 2–4 years using 30 years of individual‐level data and investigated how the age of first reproduction was correlated to lifetime reproductive success and longevity. Early reproductive success was influenced by phenotypical and demographical variables, and males that first reproduced at early ages had higher lifetime reproductive success and shorter lifespan, suggesting a long‐term survival cost of early reproduction.

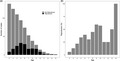

## 1 INTRODUCTION

Life‐history theory predicts diverse reproductive strategies among species and between individuals to maximize fitness (Stearns, 1992). Individuals of the same species also vary in the capacity to acquire energy, and since resources are limited in natural environments, allocation trade‐offs between fitness components are expected (Hamel et al., 2010; van Noordwijk & de Jong, 1986). Trade‐offs between growth, survival, and reproduction have been found in several species (Cox et al., 2010; Folkvord et al., 2014). For example, female red squirrels (*Tamiascirus hudsonicus*) with greater resource acquisition capacity started to reproduce earlier at the expense of decreased longevity, while females with fewer resources delayed maturity and had greater longevity (Descamps et al., 2006). The risk of dying before reproducing increases with each reproductive opportunity missed (Blomquist, 2009). Thus, the age at which an individual first allocates to reproduction may substantially influence fitness, as reported in birds (Aubry et al., 2009; Cooper et al., 2009), terrestrial mammals (Markussen et al., 2019; Martin & Festa‐Bianchet, 2012; Neuhaus et al., 2004), marine mammals (Hadley et al., 2006; Lloyd et al., 2020), fish (Swain et al., 2007), and reptiles (Bonnet et al., 2002).

Most studies of wild vertebrates testing early‐ and late‐life trade‐offs in just one sex were conducted on females (Lemaître et al., 2015). Drivers of female life‐history trade‐offs in polygynous species may not affect males, because of substantial differences in consequences of reproductive effort between sexes. While female fitness is mostly limited by forage resources, male fitness is limited by fertilization opportunities, leading to male–male competition as the main determinant of reproductive success (Bateman, 1948; Clutton‐Brock, 1988; Trivers, 1972). Little information is available about reproductive costs in male mammals, partly because allocation to competition with other males has an uncertain relationship to actual reproductive success. Energy spent competing with other males does not guarantee mating success (Festa‐Bianchet, 2012; Hamel et al., 2010; Lemaître et al., 2020; Lloyd et al., 2020; Pelletier et al., 2006). In addition, parentage assignment is challenging in wild populations because it requires genetic material from juveniles and most putative fathers (Coltman et al., 2005).

In iteroparous species, individuals in natural environment experience allocation trade‐offs between life‐history traits, such as growth pattern, age at first reproduction, lifetime reproductive success, and longevity (Stearns, 1992). Excessive allocation to reproduction during one reproductive event can decrease immediate survival (Chase, 1999) or compromise future reproduction (Nilsson & Svenssonn, 1996). On the other hand, an individual that allocates most available energy to survival is not guaranteed to reproduce in the future, especially if mortality is high or intrasexual competition increases (Bell, 1980; Chase, 1999; Wittenberger, 1979).

When the ability to acquire resources differs among individuals, the consequences of allocation to reproduction may also vary, as individual with more resources can increase allocation without being forced into trade‐offs (Hamel et al., 2010; van Noordwijk & de Jong, 1986). In polygynous systems, where a few highly competitive males can monopolize reproduction over one or a few breeding seasons (Andersson, 1994), differences in resource acquisition are likely very important. In polygynous species, male reproductive success is generally associated with dominance, which is often determined by body mass and size of secondary sexual traits (Bergeron et al., 2010; Lloyd et al., 2020; Martin et al., 2013; Pelletier & Festa‐Bianchet, 2006). When reproductive success is monopolized by a few dominant males, most males do not reproduce (Coltman et al., 2002).

In some species, young subordinate males adopt alternative mating tactics (Hogg, 1984; Pelletier et al., 2006; Willisch et al., 2012). Allocation to reproduction at early ages could have substantial consequences on male lifetime reproductive success (Bergeron et al., 2010). Those consequences could be positive if early attempts to reproduce increase experience and therefore subsequent reproductive success, or negative if competitive interactions involve a risk of injury or substantial energy costs (Bergeron et al., 2010; Weladji et al., 2008). Early allocation to reproduction can also reduce longevity through long‐term costs (Lemaître et al., 2020). For example, male ungulates that participate actively in the rut risk injuries and lose body mass, which can decrease survival probabilities year after year, thus decreasing longevity (Bergeron et al., 2010; Yoccoz et al., 2002). Because only a few studies have the necessary long‐term data on individual males, however, the causes and consequences of early reproductive success for males of polygynous species are mostly unknown. This study seeks to identify the factors influencing early male reproductive success in a polygynous species, bighorn sheep (*Ovis canadensis*), and evaluate the consequences of early reproduction for longevity and lifetime reproductive success.

Reproductive success in male bighorn sheep is strongly associated with social rank, which tends to increase with age and mass (Pelletier & Festa‐Bianchet, 2006). Starting in October, males form pre‐rut congregations and establish the annual social rank through agonistic interactions (Festa‐Bianchet, 1986; Pelletier & Festa‐Bianchet, 2004, 2006). Rutting activities also involve a risk of injury, which can decrease survival (Hogg & Forbes, 1997). During the rut, males mostly use either a tending or coursing tactic (Hogg, 1984; Hogg & Forbes, 1997). Tending is used exclusively by dominant males and consists of defending a single estrous ewe against competitors. In most days, there are between one and three females in estrous at the same time. Thus, if there is only one estrous female, the male at the top of the social rank will tend her, but if there are three estrous females at the same time, the second and third most dominant male will also use the tending tactic (Hogg, 1984). The alternative tactic, coursing, is used by subordinate males and involves attempts, often by a group of subordinates, to separate the tending male from the ewe and force a copulation (Hogg, 1984, 1987). Although the tending tactic is most efficient, about 40% of paternities are obtained by coursing males (Hogg & Forbes, 1997).

Reproductive success in males is influenced by secondary sexual traits, such as body mass, and sexual selection increases with the number of competitors (Martin et al., 2016). Body mass is a major determinant of male reproductive success in many polygynous mammals including bighorn sheep (Pelletier & Festa‐Bianchet, 2006), but the importance of body mass for young males, that are always too small to adopt the tending tactic, is unclear. It has been shown that sexual selection on body mass is present at all ages and increases with the number of competitors (Martin et al., 2016). In young males, body mass could influence the ability to participate in the rut by being more active or outcompeting other subordinates (Festa‐Bianchet, 2012; Mysterud et al., 2003). Demographic parameters are also likely to affect early reproductive success. Age structure, sex ratio, and population density are expected to affect reproductive success in ungulate males (Komers et al., 1997; Markussen et al., 2019; Newbolt et al., 2017) because male–male competition may decrease when more breeding females are available (Clutton‐Brock et al., 1997).

In this study, we explore the determinants of early reproductive success and analyze the long‐term life‐history costs of early reproduction. We first investigated how body mass, age structure, sex ratio, and population density affected reproductive success of males aged 2–4 years in a wild population at Ram Mountain, Alberta. We chose this age group because no paternities were ever assigned to males younger than 2 years and because males aged two to four are expected to exclusively use the coursing tactic, based on rut observations at Sheep River, Alberta (Pelletier et al., 2006). We predicted that body mass should increase reproductive success at all ages, because larger males should be able to sustain greater effort in coursing competition (Festa‐Bianchet, 2012). We examined the effects of age structure under the expectation that a high proportion of young males within the competitive pool decrease the effectiveness of tending males defending estrous ewes, so that young males should obtain a greater share of paternities (Bonenfant et al., 2004). A breeder sex ratio skewed toward males should increase competition for available estrous ewes, and young males may be completely excluded from reproduction (Bonenfant et al., 2004). High population density may similarly decrease the reproductive success of young adult males through an increase in competition (Mysterud et al., 2003) For example, in red deer, young males are less likely to allocate to reproduction when the level of competition is high (Mysterud et al., 2003).

We then investigated how early reproduction affects long‐term fitness by analyzing its effects on longevity and lifetime reproductive success. If early reproduction was only possible for males that had acquired substantial resources, then early reproductive success should have a positive relationship with longevity and lifetime reproductive success (Hamel et al., 2010; van Noordwijk & de Jong, 1986). If early reproductive success led to a substantial drain on body resources, however, it should have a negative relationship with longevity and lifetime reproductive success (Metcalfe & Monaghan, 2003).

## 2 MATERIALS AND METHODS

### 2.1 Study area and population

Bighorn sheep have been monitored since 1971 on Ram Mountain (52°N, 115°W), Alberta, Canada. The study area is approximately 38 km^2^, and the sheep population is mostly isolated by coniferous forests, which surround the mountain except on the North‐West side where the North Saskatchewan River separates Ram Mountain from Shunda Mountain, which harbors another small population of bighorn sheep. From late May to late September, sheep were captured in a corral trap baited with salt. Most adults were captured two to five times each summer. At each capture, body mass was measured to the nearest 250 g using a Detecto spring scale (Brooklin, NY). Repeated measurements from each individual each year allowed us to adjust mass to September 15 using individual growth curves. Linear mixed models with a restricted maximum likelihood were used to adjust mass fitted as a function of date with May 25 as day 1 (Martin & Pelletier, 2011). Adjusted mass was not estimated for individuals not captured within 50 days of September 15. All yearlings and adults were marked during our study, and more than 95% were marked as lambs (Pigeon et al., 2016). Males were marked using unique combinations of colored and numbered Allflex ear tags. Lambs were marked with numbered Ketchum metal tags (Ketchum Manufacturing) and colored strips of Safeflag plastics (Pawtucket, R.I.), which were replaced by Allflex tags at 1 year of age. The probability of detection for surviving sheep is more than 95% for males and 99% for females (Bonenfant et al., 2009).

Analyses included every male aged at least 2 years during the ruts between 1987 and 2017. Our sample thus began with rams aged at least 3 years in May 1988, when DNA sampling was initiated (Coltman et al., 2002). A male aged three in 1988 would have participated in the 1987 rut as a 2 year‐old. Of 157 males captured between 1988 and 2018, 136 had known annual reproductive success and body mass adjusted to September 15. Of those 136 males, 76 sired at least one lamb throughout their lives. Hunters could harvest males with horns of at least four‐fifths of a curl until 1995, and only full‐curl individuals from 1996 to 2011 when the hunting season was closed (Pelletier & Coltman, 2018). Of 16 shot males that were included in analyses, 11 had sired at least one lamb.

### 2.2 DNA sampling and paternity assignment

Maternity was determined by observation of suckling since 1971, but fathers were unknown until DNA analyses began in 1988. Hair, blood, or ear tissue was collected from all sheep at first capture from 1988 to 1993 and from 1997 to 2018. About 20–30 hairs including follicles and around 5 mg of ear tissue were used to extract DNA with the QIAamp tissue extraction kit (Qiagen Inc., Mississauga, Ontario). DNA was extracted from blood using a standard phenol–chloroform method. The genotyping protocol is detailed in Coltman et al. (2003) and Poissant et al. (2013). Paternity was assigned using the likelihood‐based approach from Marshall et al. (1998), and the software CERVUS was used to estimate the critical difference in log‐likelihood score for paternity assignment under a statistical confidence of 95% (Coltman et al., 2002). In 1988–2018, 770 lambs were seen, 721 were sampled for DNA and 380 were assigned to a known father. Only lambs that survived to be captured and genotyped could be assigned a father. Lambs that died before they were sampled, or were sired by immigrant males of unknown identity, were not included in subsequent analyses.

### 2.3 Statistical analyses

To analyze variables that potentially influence early reproductive success, we used generalized linear models with a binomial distribution where the response variable was the success or failure to sire at least one lamb at a given age. Variables examined were body mass adjusted to September 15, age structure, breeder sex ratio, and population density. The number of lambs caught the year following each rut was also included as a fixed effect to control for variability in siring potential.

We first compared the influence of two possible metrics of body mass: absolute mass adjusted to September 15 and mass relative to the average mass of all adult males in the population each year. Because males only compete with other males alive at the same time, we expected that a measure of relative mass would outperform absolute mass. A total of eight models per age considered body mass as fixed effect. Each was tested once with absolute and once with relative mass. We compared these two variant candidates using Akaike model selection with a ΔAICc ≥ 2 as a threshold for selection (Burnham & Anderson, 2004). We then counted the number of times each body mass measure (relative vs. absolute) was included in the best candidate model and used the mass measure with the most support in subsequent analyses.

As more than 99% of the sheep on Ram Mountain are marked, we calculated demographic variables directly. Because only males aged at least 5 years use the tending tactic (Pelletier & Festa‐Bianchet, 2006), age structure was calculated as the ratio of the number of males aged 2–4 years to the total number of males. Breeder sex ratio was the number of lactating females in the spring following the rut over the number of males aged 2 years and older alive during the rut (Martin et al., 2016). Population density was the number of sheep aged 2 years and older in June each year, the earliest time when a complete count of sheep having survived the winter was available. If an individual was not seen the following season, it was considered dead.

We considered young adult males aged two (*n* = 120), three (*n* = 96), and 4 years (*n* = 70). We built a series of models with different combinations of variables, compared them using Akaike model selection and ranked them from the lowest AICc. Model averaging was done using every model until the cumulative ΔAICc weight was 0.95 or greater. This method produces a 95% confidence set of models, or a list of models that includes the best approximating model with a certainty of 95% (Symonds & Moussalli, 2011). Adjusted standard error was included with every estimate. The explained variance was estimated using the coefficient of determination of the model with the lowest AICc. The same model was used to calculate a variance inflation factor (VIF) to quantify multicollinearity between parameters. A threshold of 3 was set as indicator of multicollinearity (Zuur et al., 2010). All statistical analyses were conducted in R (version 3.6.2).

**FIGURE 1:**
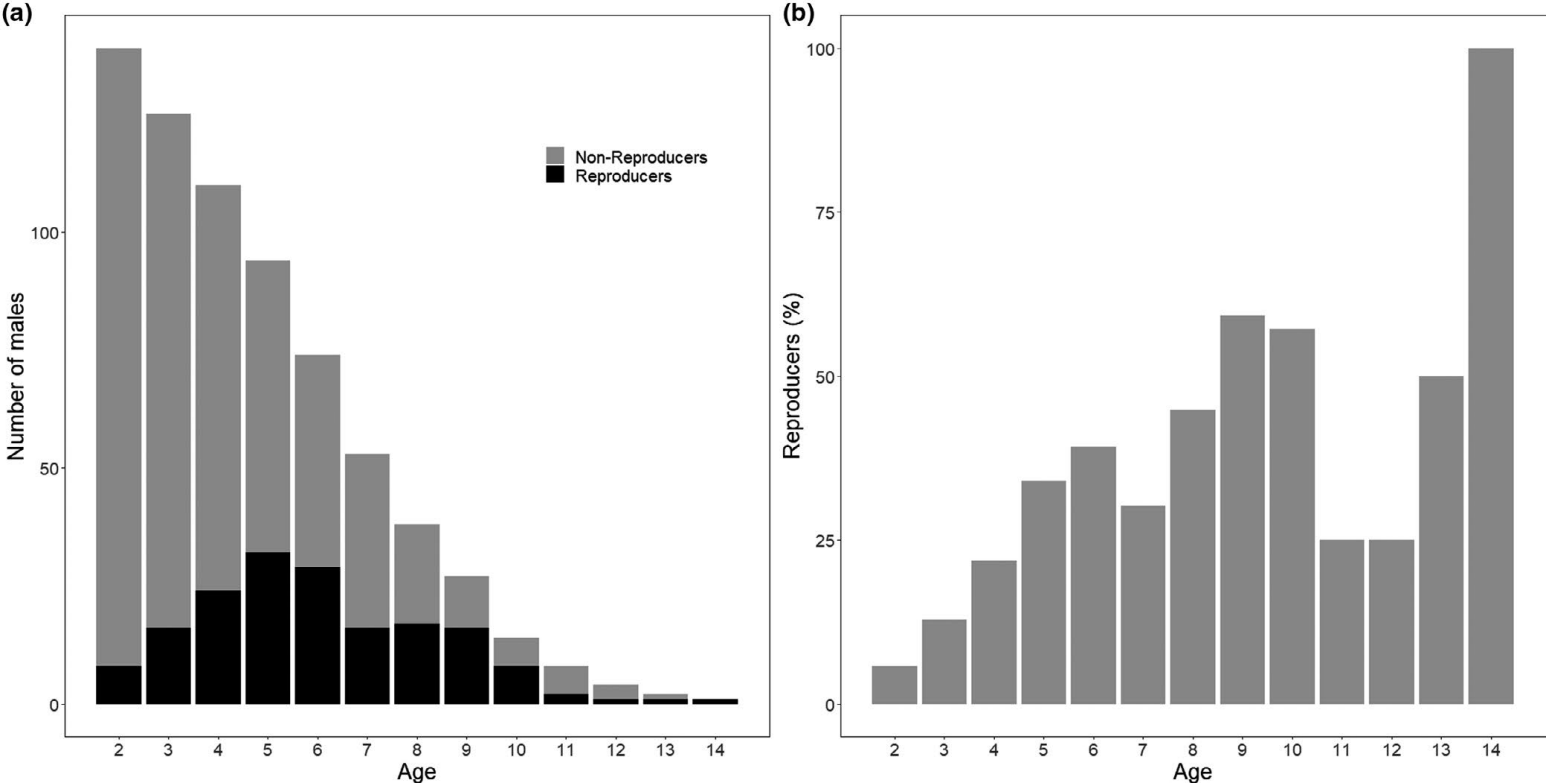
Age distribution of 141 bighorn rams aged two years and older that successfully reproduced at Ram Mountain, Alberta, ruts 1987–2017. No paternities were assigned to lamb or yearling males. (a) Reproducers and nonreproducers at each age. (b) Percentage of males that reproduced for each age

We then evaluated whether the age when the first paternity was detected affected longevity and lifetime reproductive success (LRS), considering only males known to sire at least one lamb during their lifetime (Figure 1). We did these analyses in three steps, always including body mass at 2 years as a fixed effect. We excluded 16 males shot by hunters and 14 with unknown longevity, lifetime reproductive success, or mass at 2 years, leaving a total of 51 males. We also excluded males aged 2–4 years in 1994–1996 when DNA data were not collected from lambs. LRS analyses used generalized linear models with a Poisson distribution and longevity analyses used linear models. We then repeated these analyses for LRS and longevity on a subset that included only males that first reproduced by the age of four and survived to at least 4 years of age. These criteria avoid the bias of late first reproduction being inevitably correlated with longevity, as males that die young cannot first reproduce at an advanced age. A final step compared LRS of males that first reproduced before and after 5 years of age, when the tending tactic becomes possible (Pelletier et al., 2006). Model construction and selection followed the same method used to assess the determinants of early reproduction.

## 3 RESULTS

### 3.1 Body mass candidate metrics

We first selected the best body mass variable to use in subsequent analyses, by comparing absolute mass adjusted to September 15 and adjusted mass relative to all other adult males each year. Akaike model selection showed that absolute mass was the best candidate for all models for males aged two and four years, and the best candidate for six models for 3 year‐old males. Therefore, we used absolute mass in subsequent analyses (Table A1).

### 3.2 Determinants of reproductive success between two and four years of age

**FIGURE 2:**
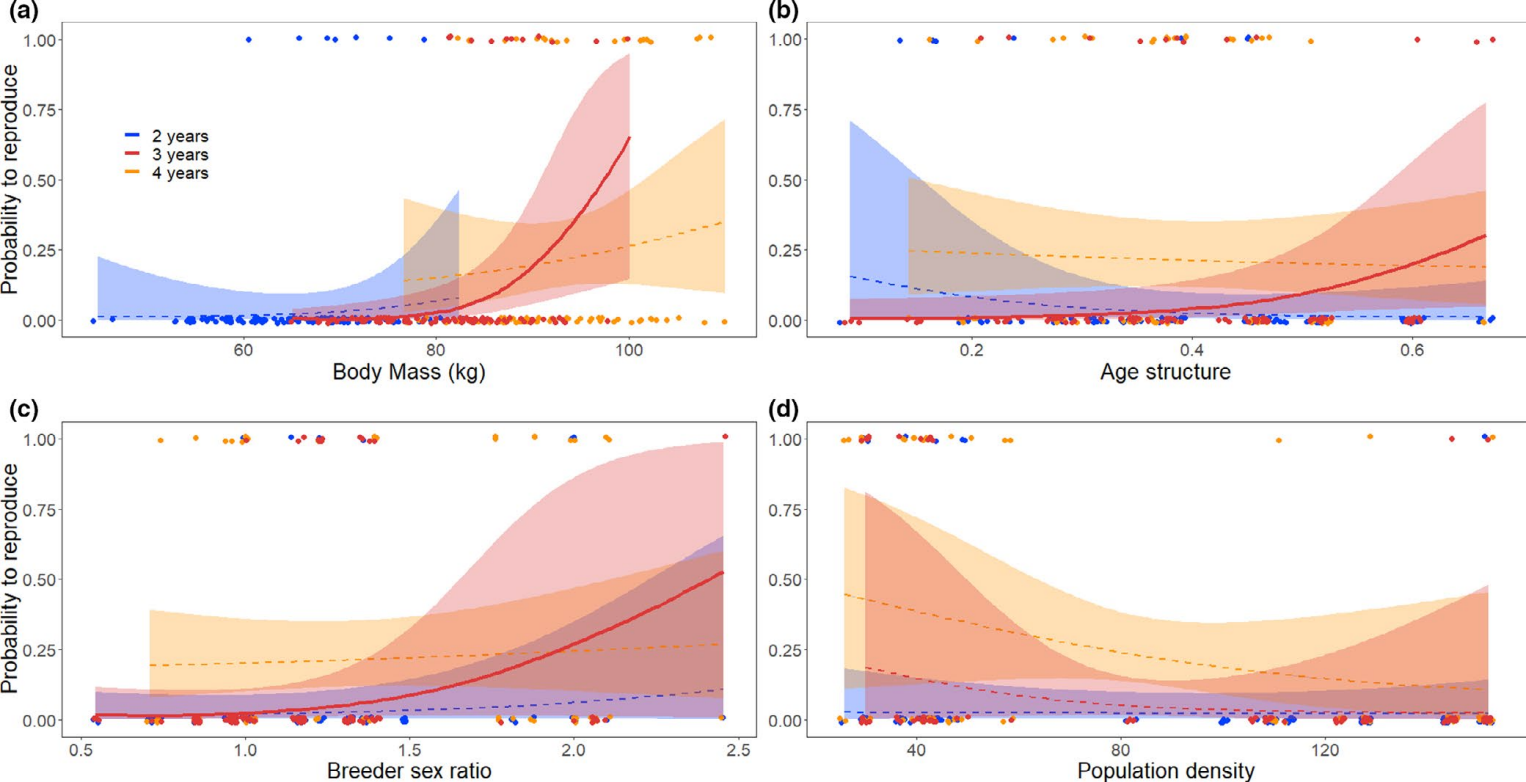
Probability to reproduce as a function of body mass, sex ratio, age structure, and population density for young bighorn sheep males at Ram Mountain, Alberta, ruts 1987–2017. Estimates were provided by model averaging presented in Tables A1‐A3. Lines indicate the estimated probability to reproduce, bold when significant and dashed when not significant. Shading represents the 95% confidence intervals, and dots are data points. Body mass (a) was adjusted to September 15 (kg); age structure (b) was the ratio between males aged two to four years and the total number of adult males; breeder sex ratio (c) was the number of lactating females in spring following the rut over the number of males aged two years and older during the rut; population density (d) includes all individuals aged two and older in June the year of the rut

We compared 16 models for each age class between two and four years (Tables A2‐A4). When the confidence interval of a coefficient did not overlap zero, we considered that parameter to have a significant effect. In total, 20% of lambs with a known father were sired by males aged 2–4 years of age. For 120 2 year‐olds (7 reproducers or 6%; Figure 1), none of the variables considered affected the probability of obtaining a paternity (Table 1). For 96 three‐year‐olds (12 reproducers or 12.5%; Figure 1), reproductive success increased in years when there was a greater proportion of young males in the population and when breeder sex ratio was more female‐biased. Heavier three‐year‐olds had a greater probability of siring a lamb than lighter ones (Table 1). Density had no effect on reproductive success. For 70 four‐year‐olds (19 reproducers or 27%; Figure 1), there was a negative, marginally nonsignificant effect on reproductive success of increasing population density (Table 1). No other variable significantly influenced reproductive success at 4 years (Figure 2).

**TABLE 1:** Estimates of the effects of body mass, age structure, sex ratio, population density, and number of lambs sampled for DNA on the reproductive success of bighorn sheep males aged two, three, and four years at Ram Mountain, Alberta, ruts 1987–2017

Fixed effect	Estimate	Adjusted *SE*	CI 2.5%	CI 97.5%
2 years				
Intercept	−5.28	6.02	−17.14	6.57
Age structure	−7.60	4.23	−15.98	0.78
Body mass	0.11	0.07	−0.03	0.26
Lambs*_t_*_+1_	−0.05	0.05	−0.14	0.04
Breeder sex ratio	1.63	1.15	−0.63	3.90
Density	0.00	0.02	−0.05	0.04
3 years				
Intercept	−23.81	8.64	−40.74	−6.88
**Age structure**	**9.09**	**3.84**	**1.56**	**16.62**
**Body mass**	**0.21**	**0.08**	**0.05**	**0.36**
Lambs*_t_*_+1_	−0.02	0.08	−0.17	0.13
**Breeder sex ratio**	**3.03**	**1.47**	**0.15**	**5.91**
Density	−0.04	0.03	−0.10	0.03
4 years				
Intercept	−2.87	4.62	−11.93	6.19
Density	−0.02	0.01	−0.05	0.00
Body mass	0.07	0.04	−0.02	0.15
Lambs*_t_*_+1_	−0.02	0.05	−0.11	0.07
Breeder sex ratio	0.74	0.86	−0.95	2.43
Age structure	−2.41	3.27	−8.82	3.99

Note

Sample sizes were 120, 96, and 70, respectively. For each age, we compared 16 models. Estimates were obtained from model averaging using a 95% AICc weight confidence set, reached by cumulating 13, 5, and 14 models for two‐, three‐, and four‐year old, respectively. The model with the lowest AICc value (AICc weight = 0.21) explained 15.5% of the observed variance at two years, 30.8% at three years (AICc weight = 0.45), and 24.3% at four years (AICc weight = 0.24). Fixed effects whose confidence interval (CI) does not overlap zero are shown in bold.

### 3.3 Long‐term effects of age at first reproduction on fitness

**FIGURE 3:**
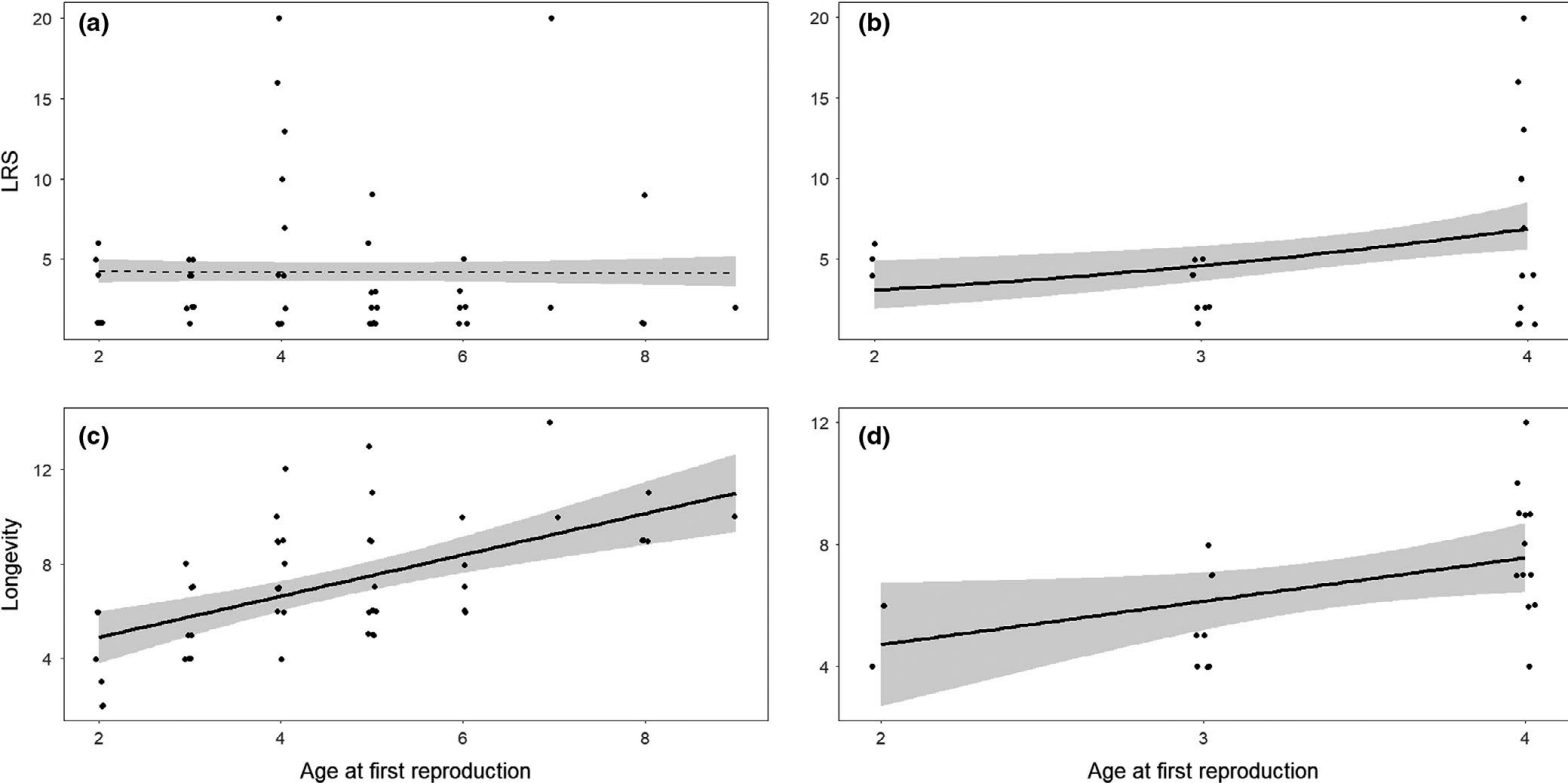
Effect of age at first reproduction (AFR) on lifetime reproductive success (LRS) and longevity for bighorn sheep males at Ram Mountain, Alberta, ruts 1987–2017. Only males known to sire at least one lamb are included. Males shot by hunters were excluded (*n* = 16). Panels (a) and (c) consider all males (*n* = 51). Panels (b) and (d) consider only 23 males that first reproduced between two and four years and survived at least four years. Figures are based on the model averaging estimates presented in Tables A4 and A6. Bold lines indicate model estimates, gray areas represent the confidence intervals (95%), and black circles are data points

The first step of this analysis considered 51 males that reproduced at least once and died naturally, comparing ten models of the effect of age at first known reproduction on lifetime reproductive success (Table A5), which was the total number of lambs assigned to a male over its lifetime. None of the variables considered had a significant effect (Table 2). We then analyzed the effect of age at first reproduction on longevity. We repeated these two analyses including only 23 males that first reproduced between two and four years of age and survived at least 4 years (Table A6). This subset analyzed exclusively the effect of reproductive success at early ages, when only coursing is available. It also avoids the bias of late first reproduction being inevitably correlated with longevity, as males that die young cannot first reproduce at an advanced age. A positive relationship between age at first reproduction and LRS was observed when considering only males aged 2–4 years (Table 2; Figure 3). We also observed a positive relationship between longevity (Tables A7‐A9) and age at first reproduction with both datasets, with early reproducers dying at a younger age (Table 3; Figure 3).

**TABLE 2:** Estimates of the effects of age at first reproduction and body mass at two years on lifetime reproductive success of bighorn sheep males at Ram Mountain, Alberta, ruts 1987–2017

Fixed effects	Estimates	Adjusted *SE*	CI 2.5%	CI 97.5%
All adults				
Intercept	1.40	0.38	0.64	2.15
AFR	−0.01	0.04	−0.09	0.07
Mass at 2 years	0.00	0.01	−0.02	0.02
2–4 years				
Intercept	0.48	0.86	−1.20	2.16
**AFR**	**0.41**	**0.15**	**0.11**	**0.70**
Mass at 2 years	−0.01	0.02	−0.04	0.02
All adults by class				
Intercept	1.94	0.63	0.71	3.17
**AFR Class (5 years+)**	**−0.48**	**0.15**	**−0.78**	**−0.17**
Mass at 2 years	−0.01	0.01	−0.03	0.01

Note

Sample size was 51 for all adults and 23 for males that first reproduced when aged two to four. Age class refers to rams that first reproduced at 2–4 years or at 5 years and older. Estimates were obtained from model averaging using the 95% confidence set method. No variance was calculated for the model including all ages since the base model had the lowest AICc value. Considering only males aged two to four, the best model explained 31.1% of the observed marginal variance (AICc weight = 0.72). When analyzing data by age class, 16% of observed marginal variance was explained (AICc weight = 0.62). Fixed effects whose confidence interval (CI) does not overlap zero are shown in bold.

**TABLE 3:** Estimates of the effects of age at first reproduction and body mass at two years on the longevity of 23 bighorn sheep males that sired at least one lamb between two and four years and survived to at least four years at Ram Mountain, Alberta, ruts 1987–2017

Fixed effect	Estimates	Adjusted *SE*	CI 2.5%	CI 97.5%
Intercept	1.70	3.30	−4.76	8.16
**AFR**	**1.55**	**0.60**	**0.38**	**2.72**
Mass at 2 years	0.01	0.07	−0.12	0.15

Note

Estimates were obtained from model averaging using the 95% confidence set method. The model with the lowest AICc value (AICc weight = 0.72) explained 25.7% of the observed marginal variance. Fixed effects where confidence interval (CI) does not overlap zero are represented in bold characters. See Tables A2‐A4 for estimates at all ages.

Finally, to compare males that successfully reproduced before or after 5 years of age, we considered three models and kept two for model averaging (Table A10). Males that first fathered a lamb when aged 2–4 years sired on average three more lambs over their lifetime than males that first reproduced at 5 years of age or older (Table 2).

## 4 DISCUSSION

Long‐term data from wild bighorn sheep show that demography and phenotype likely influence reproduction by young adult males in the Ram Mountain population. Our expectation that heavier males would be more successful than lighter males was supported only for three‐year‐olds. Reproduction by three‐year‐old males was also more likely in years with a high proportion of young males among competitors and a female‐biased breeder sex ratio. None of these predictions, however, were supported by results for males aged 2–4 years. We also found that early reproduction was associated with greater lifetime reproductive success and reduced longevity. These results suggest a long‐term survival cost of reproductive allocation early in life.

Generally, large body mass increases reproductive success in male ungulates, including bighorn sheep (Coltman et al., 2002; Markussen et al., 2019; Martin et al., 2016). For example, a positive relationship between early reproductive success and body mass was found in male moose (*Alces alces*; Markussen et al., 2019), where heavier first‐time breeders sired more calves. In our study, body mass had a significant effect on reproductive success only for three‐year‐olds. Body mass may reflect potential reproductive expenditure in ungulates (Mysterud et al., 2003, 2004) because heavier individuals can afford to lose more mass during the rut (Festa‐Bianchet et al., 1996; McElligott et al., 2003; Pelletier, 2005). Heavier three‐year‐old males may thus allocate more to reproduction than lighter ones. Behavioral observations during the rut, however, would be necessary to evaluate the correlation between effort and reproductive success. In our study, we measured reproductive success, which in males may not be strongly correlated with effort (Festa‐Bianchet, 2012). Heavier three‐year‐old males may also experience greater reproductive success by reaching an optimal body size that increases agility for the coursing tactic. Surprisingly, the positive trend between body mass and reproductive success was only significant at 3 years old. Two‐year‐old males are likely too small to compete with older males. Given that only 6% of two‐year‐olds sired a lamb, the power of this analysis was limited, and reproductive success at this age might be a stochastic event. At 4 years of age, other variables might be more relevant. Males aged 4 years are on average 14% heavier than males aged 3 years (Festa‐Bianchet et al., 1996). Perhaps the heaviest 4‐year‐old males are less successful in coursing chases due to a loss of agility, yet too small to defend estrous females through tending.

Demographic parameters also influenced the reproductive success of three‐year‐olds. In bighorn sheep, tending males defend a single estrous ewe at a time using threats, body shielding, and attacks (Hogg, 1984). An increase in proportion of young males likely leads to greater harassment of tending pairs, making the tending male less successful at securing paternity.

As expected, a female‐biased breeder sex ratio increased reproductive success for three‐year‐olds, suggesting that increased partner availability decreased competition among males (Mysterud et al., 2003). The result that neither breeder sex ratio nor male age structure affected the reproductive success of males aged 4 years supports the speculation that males of that age may be less able than 3 year‐olds to use alternative mating tactics.

Our expectation of a negative relationship between population density and early reproduction was not supported. The literature reveals that the effects of population density on male mating effort in ungulates are inconsistent (Komers et al., 1997; Mysterud et al., 2003, 2004; Yoccoz et al., 2002). In female bighorn sheep, however, density is a key driver of early reproduction (Jorgenson et al., 1993). At high density, a lower number of females produce lambs at 2 years because they allocate more to body resources to increase their own growth and survival (Jorgenson et al., 1993). A comparison of the determinants of early reproduction between sexes reinforces the idea that life‐history traits are not influenced by the same drivers in polygynous species. Density plays a major role in female early reproduction, while breeder sex ratio and age structure seem to be the most important demographic drivers in males.

Our findings are relevant to wildlife management, specifically trophy hunting, which modifies age structure and sex ratio by selectively removing dominant adult males, leading to a higher proportion of young males and a female‐biased sex ratio (Solberg et al., 2002). We found that both demographic shifts increased reproductive success in young males. An increase in young male reproductive success may relax sexual selection favoring large weapons because reproductive success of young males is mostly independent of horn or antler size (Coltman et al., 2002; Mysterud et al., 2005). Horn size is a major driver of reproductive success later in life (Coltman et al., 2002). Given that 20% of lambs were fathered by males aged between two and four, it is possible that the strength of sexual selection may be considerably lower than in mating systems where alternative mating tactics appear to be mostly unsuccessful, such as in ibex (*Capra ibex*; Willisch & Neuhaus, 2009). Hogg and Forbes (1997) reported that 44% of lamb were fathered by coursing males of any age at Sheep River (Alberta, Canada) and National Bison Range (Montana, USA).

Our study has several limitations. We could only assign paternity to lambs that survived to be captured, mostly between 3 weeks and 3 months of age. We could not measure the reproductive successes of males whose lambs died before they could be sampled. A paternal age effect on neonatal survival could bias our results, but we know of no such effect on any wild mammal. We did not observe the rut directly, and some males could have moved for the rut to another population and obtained paternity elsewhere (Hogg & Forbes, 1997; Jorgenson et al., 1993). Males aged 2–4 years, however, rarely leave their natal population for the rut (Hogg, 2000). Parental assignations confirmed the presence of one to three immigrant males during some ruts (Pigeon et al., 2016). Our calculations of breeder sex ratio, age structure, and population density during the rut are thus affected by an unknown, but likely minor, extent by the presence of these immigrant males. In addition, behavioral observations during the rut would have allowed the construction of time budgets that may better reflect individual differences in reproductive effort (Pelletier et al., 2006).

Early reproducers obtained greater lifetime reproductive success than males that reproduced for the first time later in life, but at the cost of reduced life expectancy. Assuming that early reproduction is indicative of greater early reproductive effort, we suggest that the cost to allocate more to reproduction early in life is carried throughout life, thus reducing lifespan (Bartke et al., 2001; Metcalfe & Monaghan, 2001, 2003; Rollo, 2002). Individuals may suffer survival costs if early reproduction is made possible by rapid growth in body size early in life (Metcalfe & Monaghan, 2003). In feral sheep (*Ovis aries*), castration led to increased longevity due to reduced allocation to reproductive activities (Stevenson & Bancroft, 1995), and males with scurred horns tend to live longer since they do not fight for access to ewes (Clutton‐Brock et al., 1997). Alternatively, recent definitions of sexual selection now take into account postcopulatory competition, suggesting that reproductive success does not only depend on the ability to mate, but also to compete for access to gametes. Lemaître et al. (2020) showed how allocation to sperm competition at early ages can have negative long‐term consequences. Other studies suggest that the production of a large amount of sperm or more motile spermatozoa can be costly (Thomsen et al., 2006). Possibly, young fathers allocated more resources to sperm production at the expense of body condition, leading to a shorter lifespan.

There was much variation in lifetime reproductive success and longevity among males that first reproduced at 4 years. Life‐history theory predicts that long‐lived species will modulate the age of first reproduction depending on their capacity to reach full adult size (Stearns, 1992). However, in polygynous species, male reproductive success depends mostly on the ability to outcompete other males, which varies among breeding seasons (Newbolt et al., 2017). The variation we observed could be due to a divergence in life‐history strategies. Some individuals may allocate to reproduction early in life through the coursing tactic. Others may allocate more resources to continued growth, to increase survival and possibly the chance to become a dominant tending male later in life (Coltman et al., 2002; Pelletier et al., 2006).

Our study supports the hypothesis that male reproductive success early in life mostly depends on demography and the ability to prevail against competitors (Festa‐Bianchet, 2012). Information on the reproductive success of young males in long‐lived polygynous species helps to understand how the strength and drivers of sexual selection vary with age in species with alternative mating tactics. We found that males that first reproduce at a young age have greater fitness, but may suffer a long‐term survival cost (Hayward et al., 2014). Our findings contribute to the scarce literature on life‐history trade‐offs in males for species without paternal care by providing an example of fitness costs of early reproduction. Few long‐term studies investigated survival costs of reproduction in male mammals, and to our knowledge, costs were found in only four species: Northern elephant seal (*Mirounga angustirostris*), Southern elephant seal (*Mirounga leonina*), moose, and feral sheep (Clinton & Le Boeuf, 1993; Lloyd et al., 2020; Markussen et al., 2019; Stevenson & Bancroft, 1995). Although we found a survival cost of early reproduction, we did not directly evaluate individual mating effort during the rut. Assuming that reproductive success is at least partly correlated with reproductive effort, we suggest that the survival cost originates from elevated activity during the rut and is possibly persistent over multiple years (Bergeron et al., 2010; Metcalfe & Monaghan, 2003).

## CONFLICT OF INTEREST

None of the authors have conflict of interests.

## AUTHOR CONTRIBUTIONS

**Yanny Ritchot:** Conceptualization (equal); Data curation (equal); Formal analysis (lead); Methodology (equal); Project administration (equal); Software (equal); Supervision (equal); Validation (equal); Visualization (lead); Writing‐original draft (lead); Writing‐review & editing (lead). **Marco Festa‐Bianchet:** Conceptualization (equal); Data curation (equal); Formal analysis (equal); Funding acquisition (equal); Investigation (equal); Methodology (equal); Project administration (equal); Resources (equal); Supervision (equal); Validation (equal); Visualization (equal); Writing‐original draft (equal); Writing‐review & editing (equal). **David Coltman:** Data curation (equal); Funding acquisition (equal); Software (equal); Validation (equal); Writing‐original draft (supporting); Writing‐review & editing (supporting). **Fanie Pelletier:** Conceptualization (equal); Data curation (equal); Formal analysis (equal); Funding acquisition (equal); Investigation (equal); Methodology (equal); Project administration (equal); Resources (equal); Supervision (equal); Validation (equal); Visualization (equal); Writing‐original draft (equal); Writing‐review & editing (equal).

## ETHICAL APPROVAL

The research project was approved by an affiliate of the Canadian Council on Animal Care, the Animal Care Committee of the Université de Sherbrooke (MFB2018‐1).

## Data Availability

Data are available on Dryad (https://doi.org/10.5061/dryad.7m0cfxpt5).

## APPENDIX A

**TABLE A1:** Model selection for body mass effects on reproductive success of bighorn sheep males aged 2–4 at Ram Mountain, Alberta, ruts 1987–2017

Models	K	Absolute mass		Relative mass	
		AICc	Log‐likelihood	AICc	Log‐likelihood
2 years					
Mass	3	**52.49**	−23.14	54.06	−23.93
Mass + Age structure	4	**48.4**	−20.03	51.26	−21.46
Mass + BSR	4	**49.71**	−20.68	52.58	−22.11
Mass + Density	4	**53.86**	−22.75	54.95	−23.30
Mass + Age structure + BSR	5	**49.43**	−19.35	51.8	−20.64
Mass + Age structure + Density	5	**50.34**	−19.91	52.66	−21.07
Mass + BSR + Density	5	**51.69**	−20.58	54.75	−22.11
Mass + Age structure + BSR + Density	6	**51.4**	−19.33	53.99	−20.62
3 years					
Mass	3	**63.8**	−28.77	66.46	−30.10
Mass + Age structure	4	**62.01**	−26.79	68.58	−30.07
Mass + BSR	4	61.14	−26.35	**57.99**	−24.77
Mass + Density	4	60.68	−26.12	**59.41**	−25.49
Mass + Age structure + BSR	5	**55.1**	−22.22	58.98	−24.16
Mass + Age structure + Density	5	**56.88**	−23.11	60.68	−25.07
Mass + BSR + Density	5	61.76	−25.55	**58.01**	−23.67
Mass + Age structure + BSR + Density	6	**56.08**	−21.57	58.78	−22.92
4 years					
Mass	3	**77.29**	−35.46	78.91	−36.28
Mass + Age structure	4	**76.75**	−34.07	78.85	−35.12
Mass + BSR	4	**75.53**	−33.46	78.67	−35.03
Mass + Density	4	**73.46**	−32.42	75.59	−33.49
Mass + Age structure + BSR	5	**77.2**	−33.13	79.54	−34.30
Mass + Age structure + Density	5	**75.43**	−32.25	77.15	−33.10
Mass + BSR + Density	5	**75.33**	−32.19	77.58	−32.32
Mass + Age structure + BSR + Density	6	**77.55**	−32.11	79.3	−32.98

Note

We compared models with absolute body mass and relative body mass. Sample size was 120, 96, and 70 for males aged two, three, and four years, respectively. Every model contains the number of lambs sampled for DNA in the spring following each rut. Eight models were built for every age and each was computed twice, once with absolute, and once with relative mass. For each pair of models, the one with the lowest AICc value was considered the best candidate and is highlighted in bold. We then counted the number of times each body mass variable was considered the best candidate and the one with the highest count at every age was used for subsequent analyses on early reproductive success. Mass was either body mass was adjusted to September 15 (absolute mass) or relative mass; age structure is the ratio between males aged two to four years and the total number of males; breeder sex ratio (BSR) was the number of lactating females during the spring following the rut over the number of males two years and older during the previous rut; population density includes all individuals aged two and older in June the year of the rut.

**TABLE A2:** Model selection for determinants of reproductive success of two‐year‐old males at Ram Mountain, Alberta, Canada, ruts 1987–2017

Model	K	AICc	ΔAICc	AICc weight	Log‐likelihood	Cumulative weight
Body Mass + Age structure	4	48.40	0.00	0.19	−20.03	0.19
Body mass + Age structure + Breeder sex ratio	5	49.23	0.83	0.12	−19.35	0.31
Age structure	3	49.43	1.03	0.11	−21.61	0.42
Body mass + Breeder sex ratio	4	49.71	1.31	0.10	−20.68	0.52
Age structure + Breeder sex ratio	4	50.15	1.75	0.08	−20.90	0.60
Body mass + Age structure + Density	5	50.34	1.94	0.07	−19.91	0.67
Breeder sex ratio	3	50.45	2.05	0.07	−22.12	0.74
Age structure + Density	4	50.76	2.37	0.06	−21.21	0.80
Body mass + Age structure + Breeder sex ratio + Density	6	51.40	3.00	0.04	−19.33	0.84
Body mass + Breeder sex ratio + Density	5	51.69	3.30	0.04	−20.58	0.88
Age structure + Breeder sex ratio + Density	5	52.26	3.87	0.03	−20.87	0.90
Body mass	3	52.49	4.09	0.02	−23.14	0.93
Breeder sex ratio + Density	4	52.58	4.19	0.02	−22.12	0.95
Base model	2	52.63	4.23	0.02	−24.26	0.97
Density	3	53.46	5.07	0.01	−23.63	0.99
Body mass + Density	4	53.86	5.46	0.01	−22.75	1.00

Note

Using the 95% confidence set method, model averaging was done by adding models until the cumulative AICc weight reached 0.95. Every model has the number of lambs sampled for DNA the year following the rut included as a fixed effect. The base model only contains this variable.

**TABLE A3:** Model selection for determinants of reproductive success of three‐year‐old males at Ram Mountain, Alberta, Canada, ruts 1987–2017

Model	K	AICc	ΔAICc	AICc weight	Log‐likelihood	Cumulative weight
Body Mass + Age structure + Breeder sex ratio	5	55.10	0.00	0.45	−22.22	0.45
Body mass + Age structure + Breeder sex ratio + Density	6	56.08	0.97	0.27	−21.57	0.72
Body mass + Age structure + Density	5	56.88	1.78	0.18	−23.11	0.90
Body mass + Density	4	60.68	5.58	0.03	−26.12	0.93
Body mass + Breeder sex ratio	4	61.14	6.03	0.02	−26.35	0.95
Body mass + Breeder sex ratio + Density	5	61.76	6.65	0.02	−25.55	0.97
Body mass + Age structure	4	62.01	6.91	0.01	−26.79	0.98
Body mass	3	63.80	8.70	0.01	−28.77	0.99
Age structure + Density	4	64.55	9.44	0.00	−28.05	0.99
Age structure + Breeder sex ratio + Density	5	65.60	10.49	0.00	−27.47	1.00
Density	3	65.81	10.71	0.00	−29.77	1.00
Breeder sex ratio + Density	4	67.62	12.52	0.00	−29.59	1.00
Age structure + Breeder sex ratio	5	67.75	12.64	0.00	−29.65	1.00
Breeder sex ratio	3	70.34	15.23	0.00	−32.04	1.00
Base model	2	72.87	17.76	0.00	−34.37	1.00
Age structure	3	72.87	17.76	0.00	−33.30	1.00

Note

Using the 95% confidence set method, model averaging was done by adding models until the cumulative AICc weight reached 0.95. Every model has the number of lambs sampled for DNA the year following the rut included as a fixed effect. Therefore, the base model only contains this variable.

**TABLE A4:** Model selection for determinants of reproductive success of four‐year‐old males at Ram Mountain, Alberta, Canada, ruts 1987–2017

Model	K	AICc	ΔAICc	AICc weight	Log‐likelihood	Cumulative weight
Body mass + Density	4	73.46	0.00	0.20	−32.42	0.20
Density	3	73.78	0.33	0.17	−33.71	0.38
Body mass + Breeder sex ratio + Density	5	75.33	1.87	0.08	−32.19	0.46
Body mass + Age structure + Density	5	75.43	1.98	0.08	−32.25	0.53
Body mass + Breeder sex ratio	4	75.53	2.08	0.07	−33.46	0.60
Age structure + Density	4	75.82	2.36	0.06	−33.60	0.67
Breeder sex ratio + Density	4	75.99	2.53	0.06	−33.69	0.72
Breeder sex ratio	3	76.74	3.28	0.04	−35.19	0.76
Body mass + Age structure	4	76.75	3.29	0.04	−34.07	0.80
Age structure	3	76.88	3.43	0.04	−35.26	0.84
Base model	2	76.93	3.47	0.04	−36.37	0.87
Body mass + Age structure + Breeder sex ratio	5	77.20	3.74	0.03	−33.13	0.91
Body mass	3	77.29	3.84	0.03	−35.46	0.94
Body mass + Age structure + Breeder sex ratio + Density	6	77.55	4.09	0.03	−32.11	0.96
Age structure + Breeder sex ratio + Density	5	78.13	4.68	0.02	−33.60	0.98
Age structure + Breeder sex ratio	4	78.26	4.81	0.02	−34.82	1.00

Note

Using the 95% confidence set method, model averaging was done by adding models until the cumulative AICc weight reached 0.95. Every model has the number of lambs sampled for DNA the year following the rut included as a fixed effect. Therefore, the base model only contains this variable.

**TABLE A5:** Model selection for lifetime reproductive success as a function of age at first reproduction for bighorn sheep males at Ram Mountain, Alberta, ruts 1987–2017

Models	K	AICc	ΔAICc	AICc weight	Log‐likelihood	Cumulative weight
Base model	1	344.61	0.00	0.55	−171.26	0.55
AFR	2	346.69	2.08	0.19	−171.22	0.74
Mass at 2 years	2	346.69	2.08	0.19	−171.22	0.94
AFR + Mass at 2 years	3	348.91	4.30	0.06	−171.20	1.00

Note

Using the 95% confidence set method, model averaging was done by adding models until the cumulative AICc weight was 0.95. The base model included only the intercept. AFR represents age at first reproduction and mass at 2 years is the body mass adjusted to September 15.

**TABLE A6:** Model selection for lifetime reproductive success as a function of age at first reproduction between two and four years for bighorn sheep males at Ram Mountain, Alberta, ruts 1987–2017

Models	K	AICc	ΔAICc	AICc weight	Log‐likelihood	Cumulative weight
AFR	2	156.26	0.00	0.72	−75.83	0.72
AFR + Mass at 2 years	3	158.47	2.20	0.24	−75.60	0.96
Base model	1	163.11	6.85	0.02	−80.46	0.99
Mass at 2 years	2	164.24	7.98	0.01	−79.82	1.00

Note

Using the 95% confidence set method, model averaging was done by adding models until the cumulative AICc weight was 0.95. The base model included only the intercept. AFR represents age at first reproduction and mass at 2 years is the body mass adjusted to September 15.

**TABLE A7:** Model selection for longevity as a function of age at first reproduction and body mass at two years for bighorn sheep males

Models	K	AICc	ΔAICc	AICc weight	Log‐likelihood	Cumulative weight
AFR	2	227.78	0.00	0.68	−110.64	0.68
AFR + Mass at 2 years	3	229.32	1.54	0.32	−110.23	1.00
Base	1	247.71	19.93	0.00	−121.73	1.00

Note

Using the 95% confidence set method, model averaging was done by adding models until we had a cumulative AICc weight of 0.95. The base model included only the intercept. AFR represents age at first reproduction and mass at 2 years is the body mass adjusted to September 15.

**TABLE A8:** Model selection for longevity as a function of age at first reproduction between two and four years and body mass at two years

Models	K	AICc	ΔAICc	AICc weight	Log‐likelihood	Cumulative weight
AFR	2	99.86	0.00	0.75	−46.30	0.75
AFR + Mass at 2 years	3	102.77	2.91	0.17	−46.27	0.92
Base	1	104.32	4.47	0.08	−49.86	1.00

Note

Using the 95% confidence set method, model averaging was done by adding models until we had a cumulative AICc weight of 0.95. The base model included only the intercept. AFR represents age at first reproduction and mass at 2 years is the body mass adjusted to September 15.

**TABLE A9:** Effects of age at first reproduction and body mass at two years on the longevity of bighorn sheep males at Ram Mountain, Alberta

Fixed effect	K	Estimates	Adjusted *SE*	CI 2.5%	CI 97.5%
Base model	1	4.01	2.57	−1.03	9.06
**AFR**	2	**0.87**	**0.18**	**0.51**	**1.24**
Mass at 2 years	2	−0.04	0.05	−0.13	0.05

Note

Estimates were obtained from model averaging using the 95% confidence set method. Sample size was 51. The model with the lowest AICc value (AICc weight = 0.72) explained 34.8% of the observed marginal variance. Fixed effects where confidence interval (CI) does not overlap zero are represented in bold characters. The base model included only the intercept. AFR represents age at first reproduction and mass at 2 years is the body mass adjusted to September 15.

**TABLE A10:** Model selection for lifetime reproductive success as a function of early or late age at first reproduction for bighorn sheep males at Ram Mountain, Alberta, ruts 1987–2017

Models	K	AICc	ΔAICc	AICc weight	Log‐likelihood	Cumulative weight
AFR Class	2	336.36	0.00	0.62	−166.06	0.62
AFR Class + Mass	3	337.38	1.02	0.37	−165.43	0.99
Base model	1	344.61	8.24	0.01	−171.26	1.00

Note

AFR class divides males that reproduced for the first time before five years of age from those that reproduced after. Using the 95% confidence set method, model averaging was done by adding models until we had a cumulative AICc weight of 0.95. The base model included only the intercept. AFR represents age at first reproduction.
